# Tick salivary proteins metalloprotease and allergen-like p23 are associated with response to glycan α-Gal and mycobacterium infection

**DOI:** 10.1038/s41598-025-93031-3

**Published:** 2025-03-14

**Authors:** Rita Vaz-Rodrigues, Lorena Mazuecos, Marinela Contreras, Almudena González-García, Marta Rafael, Margarita Villar, José de la Fuente

**Affiliations:** 1 Institute for Game and Wildlife Research, SaBio, IREC-CSIC-UCLM-JCCM, Ronda de Toledo 12, 13005 Ciudad Real, Ciudad Real, Spain; 2 Biochemistry Section, Department of Inorganic, Organic Chemistry and Biochemistry, Faculty of Sciences and Chemical Technologies, University of Castilla-La Mancha, Ave. Camilo José Cela 10, Ciudad Real, 13071 Spain; 3 Department of Veterinary Pathobiology, Center for Veterinary Health Sciences, Oklahoma State University, Stillwater, OK 74078 USA; 4 José de la Fuente, SaBioInstituto de Investigación en Recursos Cinegéticos IREC-CSIC-UCLM-JCCM, Ronda de Toledo 12, 13005 Ciudad Real, Ciudad Real, Spain

**Keywords:** Allergy, α-Gal syndrome, Saliva, Tick, Tuberculosis, Zebrafish, Immunology, Diseases, Pathogenesis

## Abstract

The alpha-Gal syndrome (AGS) evolved as a catastrophic selection associated with anti-α-Gal IgM/IgG protective response against pathogen infection and tick-borne food allergy caused by IgE-type antibodies against this glycan present in glycoproteins and glycolipids from mammalian meat and derived products. The immune response to α-Gal is modulated by tick salivary proteins with and without α-Gal modifications in combination with tick saliva non-protein fraction. Herein, we characterized the role of tick salivary proteins, metalloprotease and allergen-like p23 in AGS and protection against tuberculosis in the AGS zebrafish animal model. Metalloprotease and p23 are involved in allergic reactions after mammalian meat consumption through upregulation of pro-inflammatory protein-coding genes *prkdc*, *tlr2*, *tnfα* and *il1b*. Challenge with *Mycobacterium marinum* activated Th1-mediated immune protective response with reduced pathogen infection, ameliorating Th2-associated allergic reactions associated with AGS. These results highlight molecular mechanisms modulated by tick proteins in response to α-Gal and provide insights to reduce AGS impact on human health.

## Introduction

The α-Gal syndrome (AGS) is associated with allergic reactions to tick bites and consumption of mammalian meat and pharmaceuticals containing glycan Galα1-3Galβ1-4GlcNAc-R (α-Gal) modifications in proteins and lipids^1–4^. The initial IgE sensitization is associated to bites from hard-bodied ticks such as *Amblyomma americanum*, *Ixodes ricinus*, *Ixodes holocyclus* and *Haemaphysalis longicornis* with α-Gal-containing glycoproteins and glycolipids^3,5–9^. Despite advances in the study of AGS clinical symptoms and patient’s B-cell responses, the immunogenic modulators and immune-mediated mechanisms of AGS have not been fully characterized^3,10–12^.

Hominids evolved with catastrophic selection events including the inability to synthesize α-Gal and thus the capacity to produce IgM/IgG antibodies against this molecule and activate immune mechanisms with protective capacity against pathogens containing or not this glycan modification^4,13–18^. Accordingly, immunization with α-Gal and probiotics with α-Gal content burst protective immune responses against infection by pathogens such as mycobacteria causing tuberculosis^19,20^.

Zebrafish (*Danio rerio*) has been established as an animal model for the study of AGS^21,22^ and protective immune-mediated mechanisms against tuberculosis caused by *Mycobacterium marinum*^19,20^. In a recent study, zebrafish treated with tick saliva and saliva protein fractions combined with non-protein fraction when compared to PBS-treated controls presented a higher incidence of symptoms associated with AGS such as hemorrhagic type allergic reactions, abnormal behavioral patterns, and mortality^22^. Furthermore, the proteomics characterization of tick saliva protein fractions identified metalloprotease and allergen-like p23 as tick salivary proteins with possible functional implications in response to α-Gal^22^. The results derived from these studies supporting the use of the zebrafish model for the study of AGS include the production of anti-α-Gal IgM antibodies, allergic reactions, abnormal behavior patterns and feeding in response to tick saliva and salivary biogenic substances and mammalian meat consumption, association of allergic reactions with tissue-specific TLR-mediated response, IL-4 and basophils, inhibition of pathways associated with adrenergic signaling in cardiomyocytes, and heart and muscle contraction in response to α-Gal sensitization, and the role of tick salivary proteins and lipids without α-Gal modifications in AGS. In contrast to the antibody isotypes found in humans and mice (IgD, IgM, IgA, IgG, IgE), zebrafish produce only functionally equivalent IgZ and IgT associated with mucosal immunity, and IgM for the other immune functions^23^.

To address this hypothesis, herein we characterized in the zebrafish animal model the role of tick salivary proteins, metalloprotease (MET; UniProt ID: A0A0K8RCY8) and allergen-like p23 (p23; UniProt ID: A0A0K8RKR7), in the symptomatology and immune-mediated mechanisms associated with AGS. Additionally, the interaction between these proteins and mycobacterial infection was evaluated. The results provided information to advance in the immune response to glycan α-Gal with implications in the diagnosis, treatment, and prevention of the AGS.

## Results

### Tick salivary proteins metalloprotease and allergen-like p23 are involved in allergic reactions to mammalian meat consumption in the zebrafish model of AGS

**Fig. 1:**
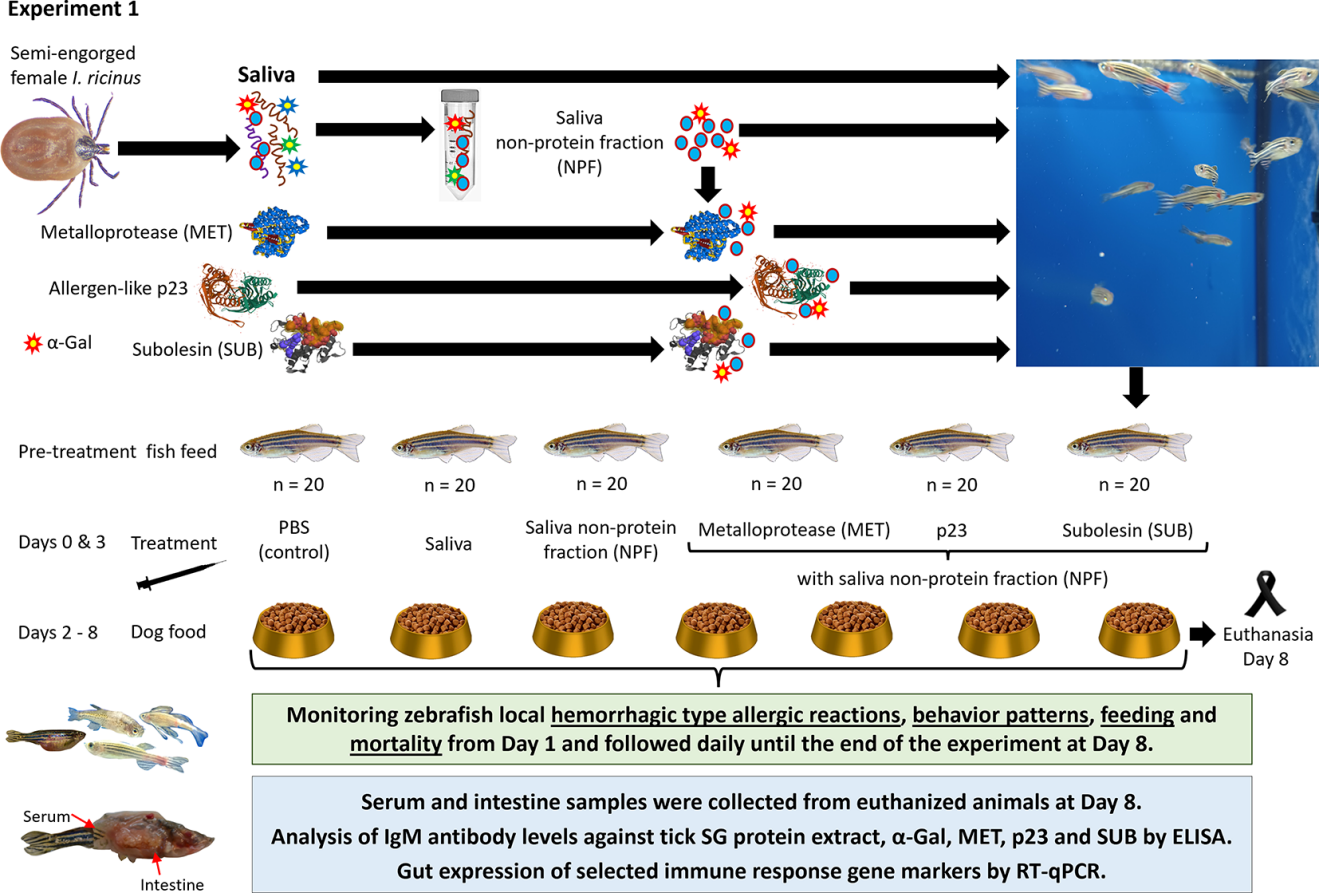
Experiment 1 Experimental design to characterize the role of tick salivary proteins in allergic reactions to mammalian meat consumption in the zebrafish model of the α-Gal syndrome (AGS). Saliva from semi-engorged *Ixodes ricinus* female ticks was collected and used in this experiment. Wild type adult AB strain zebrafish (20 animals/group) were treated with PBS, saliva, saliva non-protein fraction (NPF) alone and combined with recombinant tick proteins metalloprotease (MET), allergen-like p23 (p23) and, as control, Subolesin (SUB). Zebrafish were kept on fish feed during pre-treatment and until Day 2. Zebrafish were injected with each treatment at Days 0 and 3, and from Day 2 and until the end of the experiment at Day 8 fish were fed with dog food containing mammalian meat. Zebrafish local hemorrhagic type allergic reactions, abnormal behavior patterns and feeding and accumulated mortality were examined from Day 1 and followed daily until the end of the experiment at Day 8. After fish euthanasia, serum was collected individually to determine IgM antibody titers against tick salivary glands (SG) protein extract, α-Gal and tick proteins. Intestine samples were collected for expression analysis of selected immune response and allergy gene markers by RT-qPCR.

**Fig. 2:**
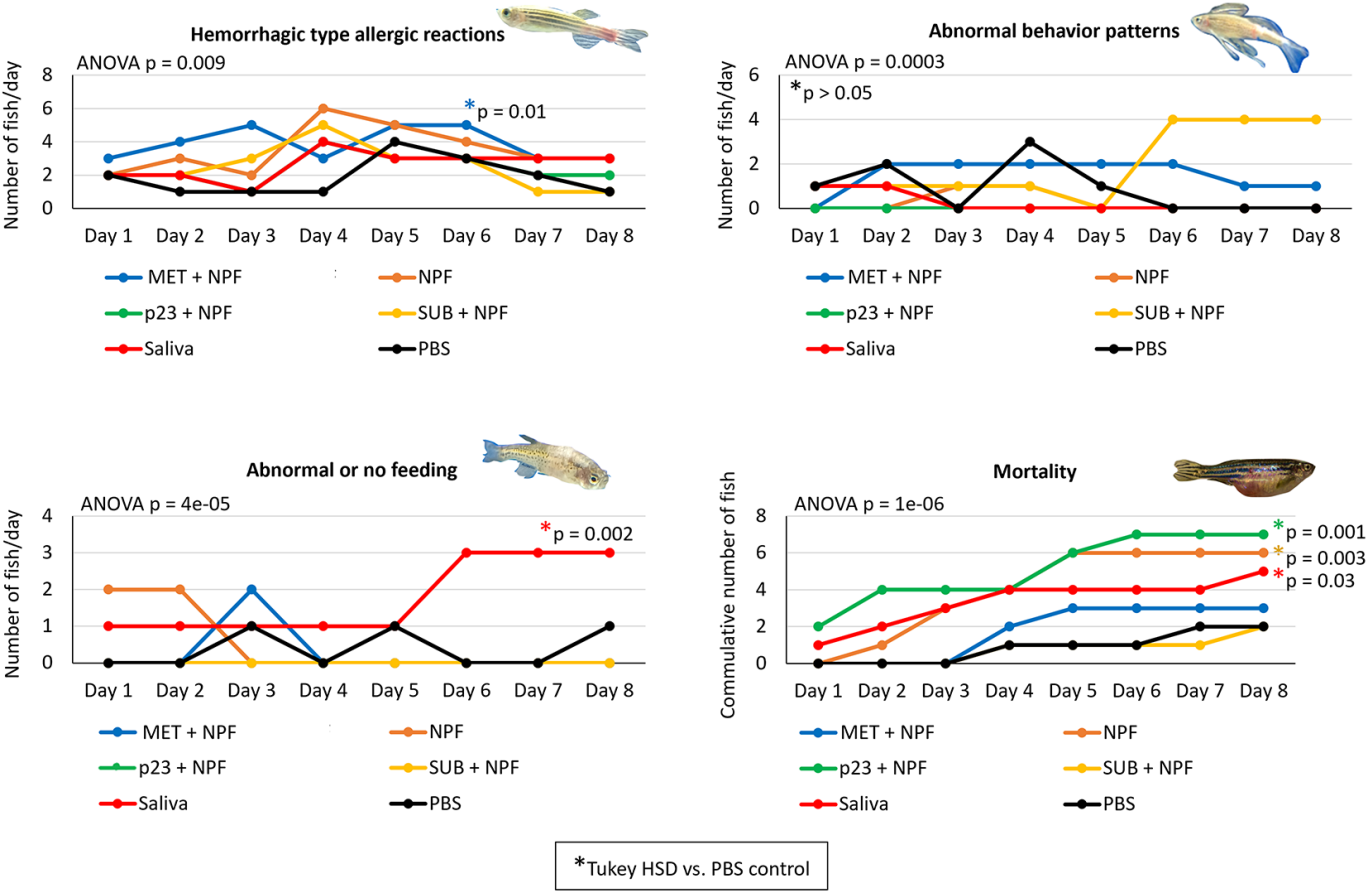
Allergic reactions to mammalian meat consumption in zebrafish treated with tick salivary proteins. Experiment 1. Zebrafish were treated with recombinant tick salivary proteins, metalloprotease (MET) or allergen-like p23 (p23), in combination with saliva non-protein fraction (NPF) and in comparison, with tick saliva, saliva NPF, Subolesin (SUB)-NPF and PBS. Zebrafish were examined daily and the incidence of hemorrhagic type allergic reactions, abnormal behavior and feeding patterns and cumulative mortality were compared between treatments by one-way ANOVA test with post-hoc Tukey HSD test (*p* < 0.05; *n* = 13–20 biological replicates). Significant differences between treatments and PBS control are shown with post-hoc Tukey HSD p-values. Color of the asterisk of significant differences are associated with treatment line.

The Experiment 1 was designed to characterize the effect of zebrafish treatment with tick MET, p23 and control Subolesin (SUB, Q4VRW2) combined with saliva non-protein fraction (NPF) (Fig. 1). Treatment with tick saliva resulted in significant higher incidence of abnormal feeding (*p* = 0.002) and mortality (*p* = 0.03) when compared to PBS-treated control (Fig. 2). Treatment with tick saliva recombinant proteins in combination with NPF to mimic saliva resulted in significantly higher incidence of hemorrhagic type allergic reactions (MET; *p* = 0.01) and mortality (p23; *p* = 0.001), the latest also associated with NPF (*p* = 0.003) (Fig. 2). As expected, treatment with tick intracellular regulatory protein SUB in combination with NPF did not cause any allergic reactions in zebrafish.

### Characterization of IgM antibody titers in zebrafish treated with tick salivary biomolecules

**Fig. 3:**
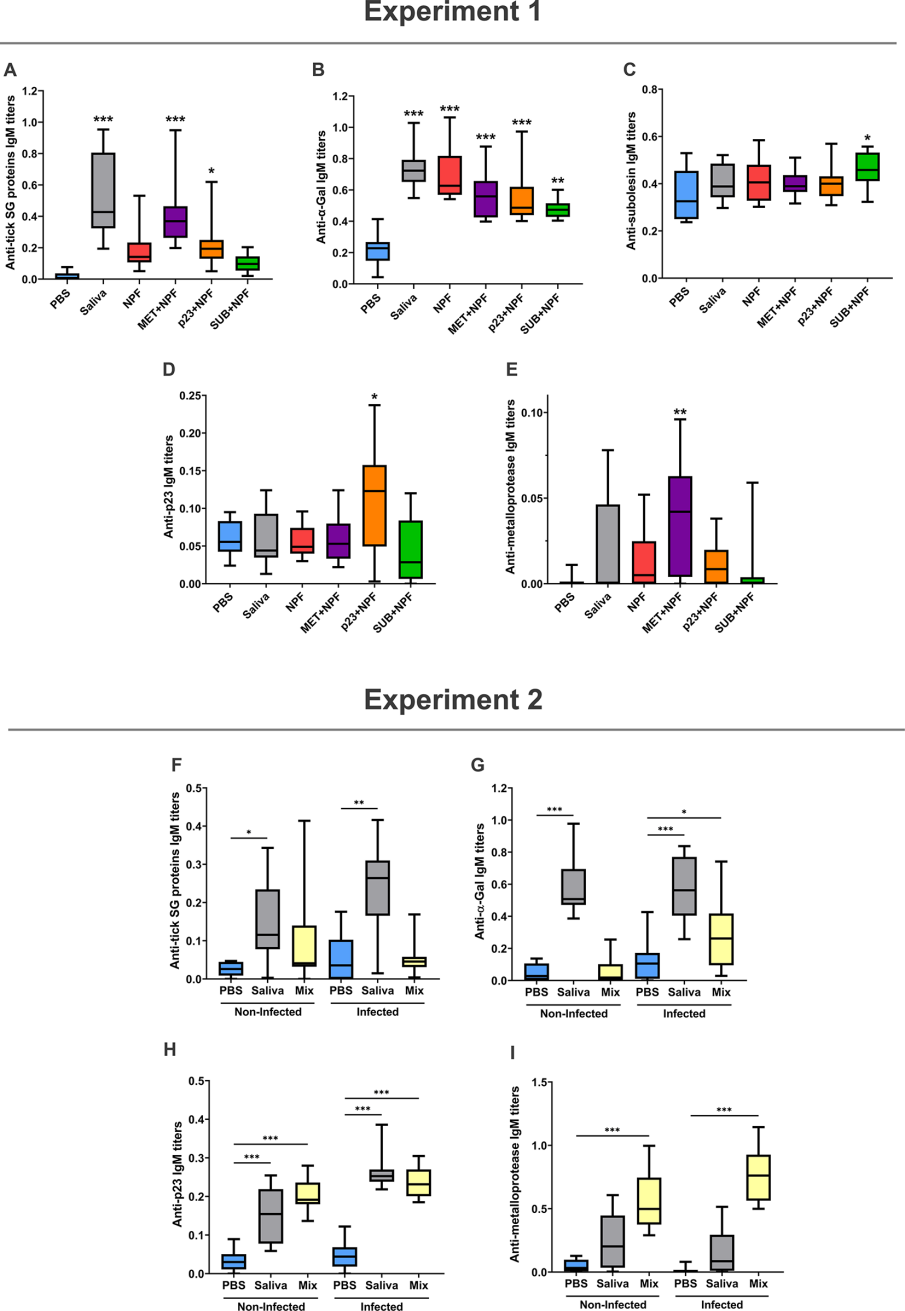
Characterization of IgM antibody titers in zebrafish AGS model treated with tick salivary proteins. Experiment 1. Zebrafish were treated with recombinant tick salivary proteins, metalloprotease (MET) or allergen-like p23 (p23), in combination with saliva non-protein fraction (NPF) and, in comparison with tick saliva, saliva NPF, Subolesin (SUB)-NPF and PBS. (**A**) IgM antibody titers against tick salivary gland (SG) protein extract. (**B**) IgM antibody titers against α-Gal. (**C**) IgM antibody titers against SUB. (**D**) IgM antibody titers against tick protein p23. (**E**) IgM antibody titers against tick protein MET. Experiment 2. Zebrafish were treated with a combination (Mix) that included both recombinant tick salivary proteins, MET and p23, with NPF and in comparison, with tick saliva, and PBS. (**F**) IgM antibody titers against tick SG protein extract. (**G**) IgM antibody titers against α-Gal. (**H**) IgM antibody titers against tick protein p23. (I) IgM antibody titers against tick protein MET. Significant differences between treatments and PBS control are shown with post-hoc Pairwise Mann–Whitney p-values (* *p* < 0.05, ** *p* < 0.01, *** *p* < 0.001).

In Experiment 1, zebrafish serological analysis of IgM antibodies against tick salivary gland protein extract revealed higher titers in groups treated with tick saliva (*p* < 0.001) and recombinant tick proteins, MET (*p* < 0.001) or p23 (*p* < 0.05) combined with NPF when compared to PBS control group (Fig. 3A). Anti-α-Gal IgM antibodies were detected in all treatment groups (tick saliva, NPF, MET + NPF, p23 + NPF and SUB + NPF) and significantly higher when compared to PBS control (*p* < 0.01, Fig. 3B). Anti-SUB IgM antibody titers were significantly higher only in SUB-treated zebrafish (*p* < 0.05, Fig. 3C). IgM antibody levels against recombinant tick proteins, p23 and MET were higher in response to treatment with these proteins combined with NPF (*p* < 0.05 for p23, Fig. 3D and *p* < 0.01 for MET, Fig. 3E) in comparison to the PBS group.

### Infection with *M. marinum* reduces the risks of allergic reactions to mammalian meat consumption in the zebrafish model of AGS

**Fig. 4:**
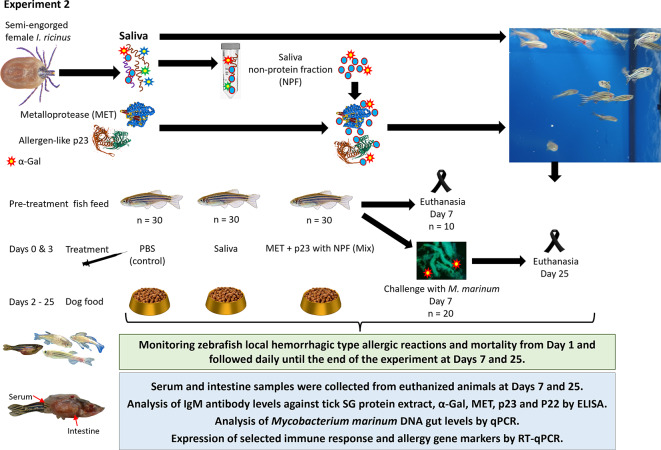
Experiment 2: Experimental design to evaluate the role of combined treatment with tick salivary proteins and fish tuberculosis. Saliva from semi-engorged *I. ricinus* female ticks was collected and used in this experiment. Wild type adult AB strain zebrafish (30 animals/group) were treated with PBS, saliva, and combined (Mix) tick salivary proteins metalloprotease (MET) + allergen-like p23 (p23) with saliva non-protein fraction (NPF). Zebrafish were kept on fish feed during pre-treatment and until Day 2. Zebrafish were injected via IM with each treatment at Days 0 and 3, and from Day 2 and until the end of the experiment at Days 7 and 25 fish were fed with dog food containing mammalian meat. At Day 7, 10 animals/group were euthanized by prolonged immersion using an overdose of tricaine methane sulfonate (MS222, 200–300 mg/l). The rest of the animals (*n* = 20) were challenged with *M. marinum* and maintained until Day 25 in which they were sacrificed. Zebrafish local hemorrhagic type allergic reactions and accumulated mortality were examined from Day 1 and followed daily until the end of the experiment at Days 7 or 25. After fish euthanasia, serum was collected individually to determine IgM antibody titers against tick salivary glands (SG) protein extract, α-Gal, tick proteins and the immunopurified subcomplex protein P22. Intestine samples were collected for expression analysis of selected immune response and allergy gene markers by RT-qPCR and the analysis of *M. marinum* DNA levels by qPCR.

**Fig. 5:**
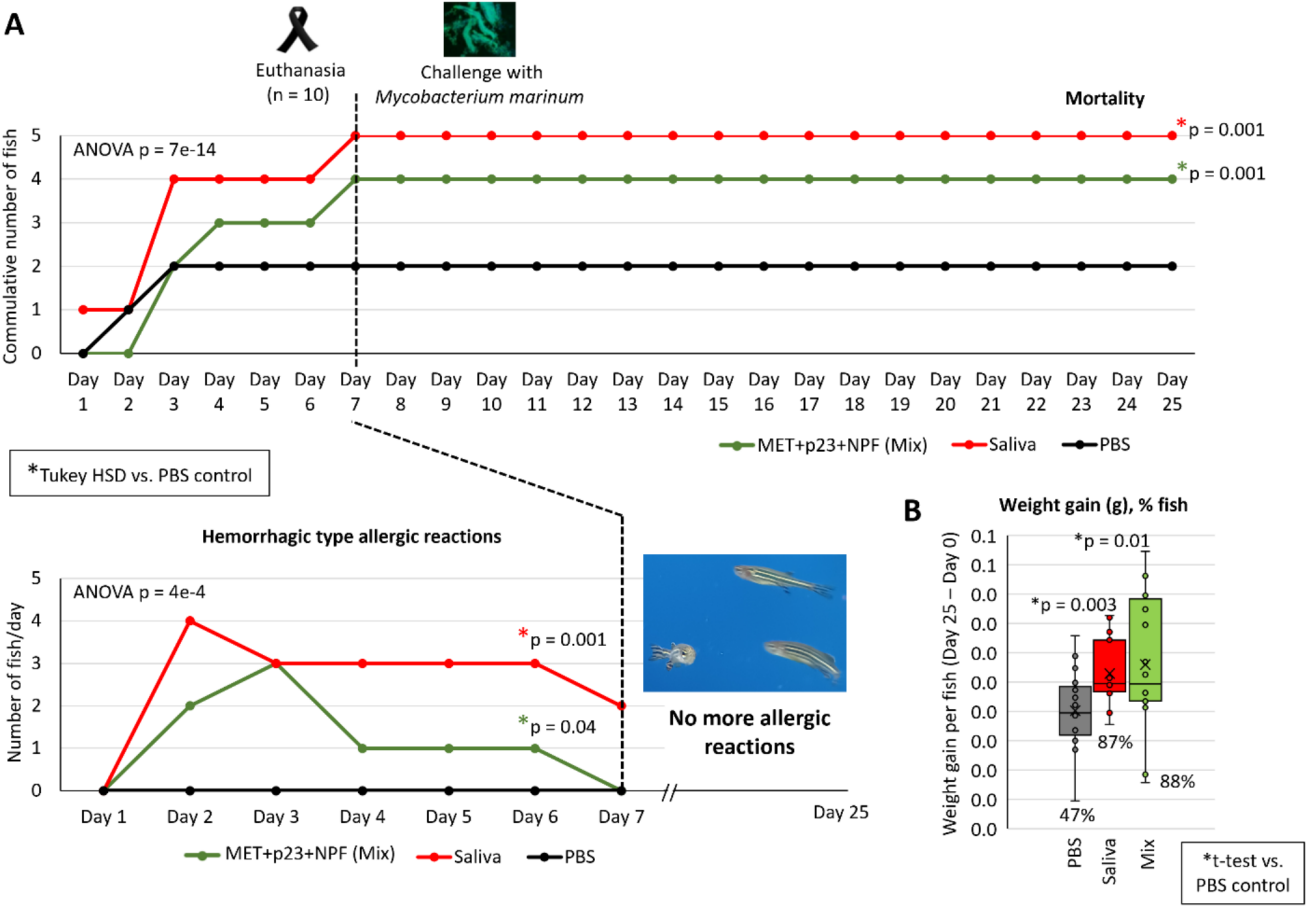
Allergic reactions to mammalian meat consumption in zebrafish treated with tick salivary proteins and challenged with *M. marinum*. Experiment 2. Zebrafish were treated with both recombinant tick salivary proteins, metalloprotease (MET) and allergen-like p23 (p23), combined with saliva non-protein fraction (NPF) (Mix) and in comparison, with tick saliva, and PBS (control). Zebrafish (*n* = 10) were euthanized at Day 7 and the rest (*n* = 20) were challenged with *M. marinum* and euthanized at Day 25. (**A**) Zebrafish were examined daily, and the incidence of hemorrhagic type allergic reactions and mortality were compared between treatments by one-way ANOVA test with post-hoc Tukey HSD test (*p* < 0.05; *n* = 13–20 biological replicates). Significant differences between treatments and PBS control are shown with post-hoc Tukey HSD p-values. (**B**) Zebrafish weight was evaluated at Day 0 and at Day 25 for each animal to calculate weight gain per fish (grams, Day 25 – Day 0) and the percentage (%) of fish showing weight gain on each group. The results were compared with PBS-treated group by Student’s t-test (*p* < 0.05; *n* = 15–17 biological replicates).

The Experiment 2 was designed to evaluate the effect of *M. marinum* on zebrafish exposed to salivary proteins and NPF (Fig. 4). In agreement with Experiment 1, treatment with tick saliva increased mortality when compared with control zebrafish treated with PBS (*p* = 0.001; Fig. 5A). The incidence of mortality was also higher in zebrafish treated with combined MET, p23 and NPF (Mix, *p* = 0.001; Fig. 5A). Treatment of zebrafish with tick saliva (*p* = 0.001) or Mix (*p* = 0.04) also increased the incidence of hemorrhagic type allergic reactions when compared to PBS control (Fig. 5A). However, after infection with *M. marinum*, the incidence of mortality and hemorrhagic type allergic reactions was not affected in any of the groups (Fig. 5A). These results suggested a protective effect of infection by *M. marinum* on AGS in response to tick salivary components and mammalian feed consumption in the zebrafish model. In agreement with these findings, body weight significantly increased after 25 days of trial in zebrafish challenged with *M. marinum* and treated with Mix (*p* = 0.01; weight gain in 88% fishes) or tick saliva (*p* = 0.003; weight gain in 87% fishes) when compared to PBS-treated control (weight gain on only 47% fishes) (Fig. 5B). In Experiment 1 with only 8 days trial (Fig. 1), zebrafish weight did not show any significant increase in any of the experimental groups (*p* > 0.05; for all zebrafish, 0.33 ± 0.06 g at Day 0 vs. 0.47 ± 0.07 ay Day 8).

### Treatment with tick salivary biomolecules in the zebrafish model of AGS reduces *M. marinum* gut infection levels and risk of tuberculosis

**Fig. 6:**
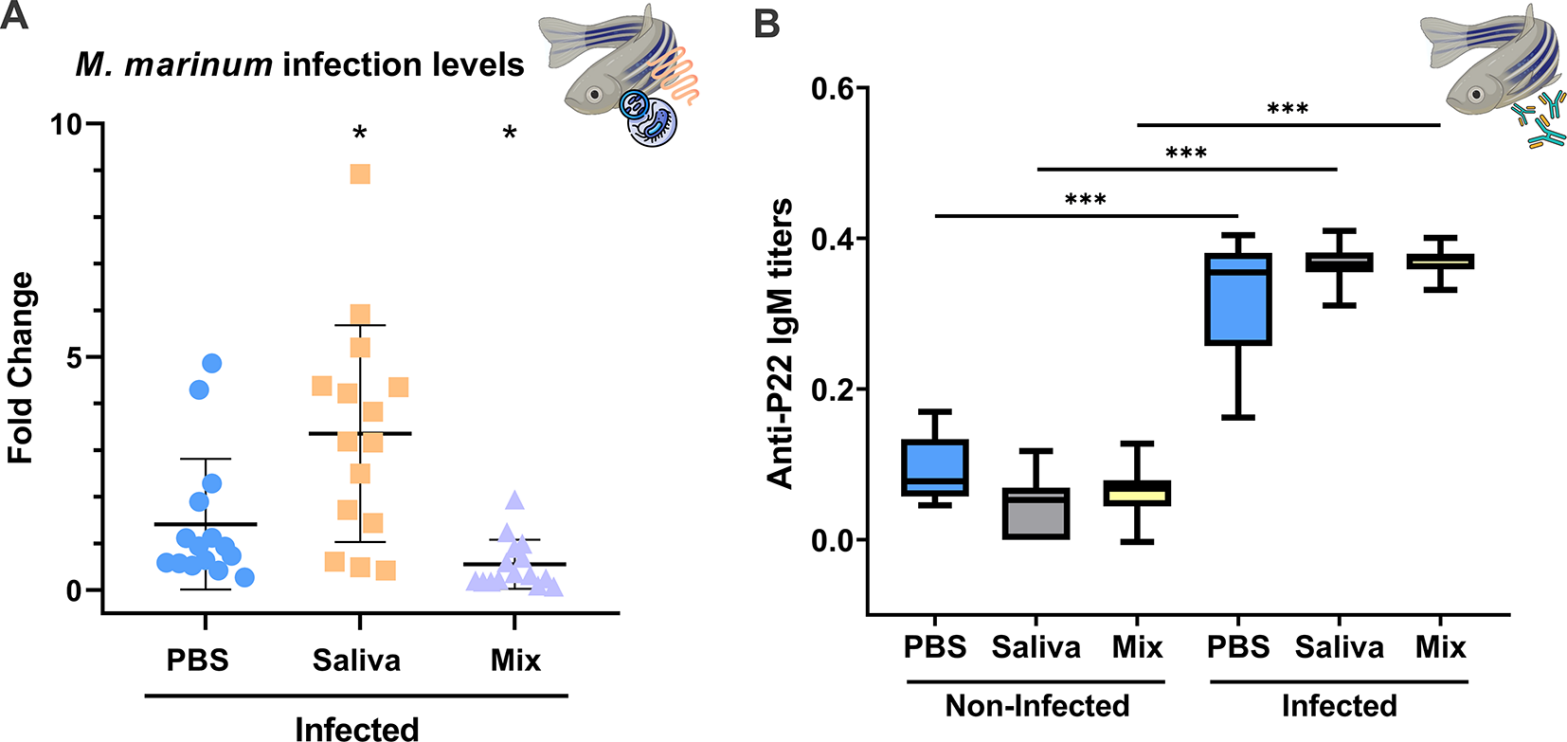
Quantification of zebrafish gut infection and antibody levels in response to *M. marinum* infection. Zebrafish were treated with a combination of MET, p23 and NPF (Mix) tick salivary biomolecules and compared with PBS control group. (**A**) Infection levels were quantified by qPCR using the 16 S ribosomal RNA (*16 S rRNA*) gene fragment. (**B**) IgM antibody titers against *M. bovis* P22 protein complex were determined by ELISA. Significant differences between treatments and PBS control are shown with post-hoc Pairwise Mann–Whitney p-values (* *p* < 0.05, ** *p* < 0.01, *** *p* < 0.001).

Zebrafish treated with Mix, *I. ricinus* tick saliva, and PBS control showed different levels of *M. marinum* infection levels in fish gut (Fig. 6A). Significantly higher infection levels were observed in the group treated with tick saliva (median Fold Change [FC] = 3.192) when compared to PBS (*p* < 0.05, FC = 0.933) and the Mix (*p* < 0.001, FC = 0.325) groups. Interestingly, the Mix treatment group presented lower *M. marinum* infection levels when compared to the PBS control group (*p* < 0.05). Anti-P22 antibodies in response to *M. marinum* infection were detected in sera from infected zebrafish with significantly higher titers when compared with non-infected animals (*p* < 0.001, Fig. 6B). However, not significant differences were observed between infected groups, suggesting that, although *M. marinum* infection levels were lower in zebrafish treated with Mix tick biomolecules (Fig. 6A), the protective response was not antibody driven.

### Characterization of IgM antibody titers in zebrafish treated with tick salivary biomolecules and infected with *M. marinum*

In Experiment 2, anti-tick salivary gland protein extract IgM antibody titers were significantly higher in both *M. marinum* infected (*p* < 0.01) and non-infected (*p* < 0.05) groups that were treated with tick saliva when compared to the PBS control group (Fig. 3F). Anti-α-Gal IgM antibodies were detected in *M. marinum* infected and non-infected groups treated with tick saliva and significantly higher when compared to PBS control group (*p* < 0.001, Fig. 3G). However, anti-α-Gal IgM titers were not higher in the group treated with Mix p23, MET, NPF and then infected with *M. marinum* (*p* < 0.05, Fig. 3G). Treatment groups that were injected with tick saliva or the Mix of p23, MET and NPF showed higher levels of IgM antibodies against allergen-like p23 (*p* < 0.001, Fig. 3H) not associated with *M. marinum* infection. However, anti- MET IgM antibody levels were only higher in Mix p23, MET and NPF treatment group when compared to PBS (*p* < 0.001), without significant differences in the tick saliva treated group (*p* > 0.05, Fig. 3I).

### Expression levels of biomarkers associated with immune and allergic response

**Fig. 7:**
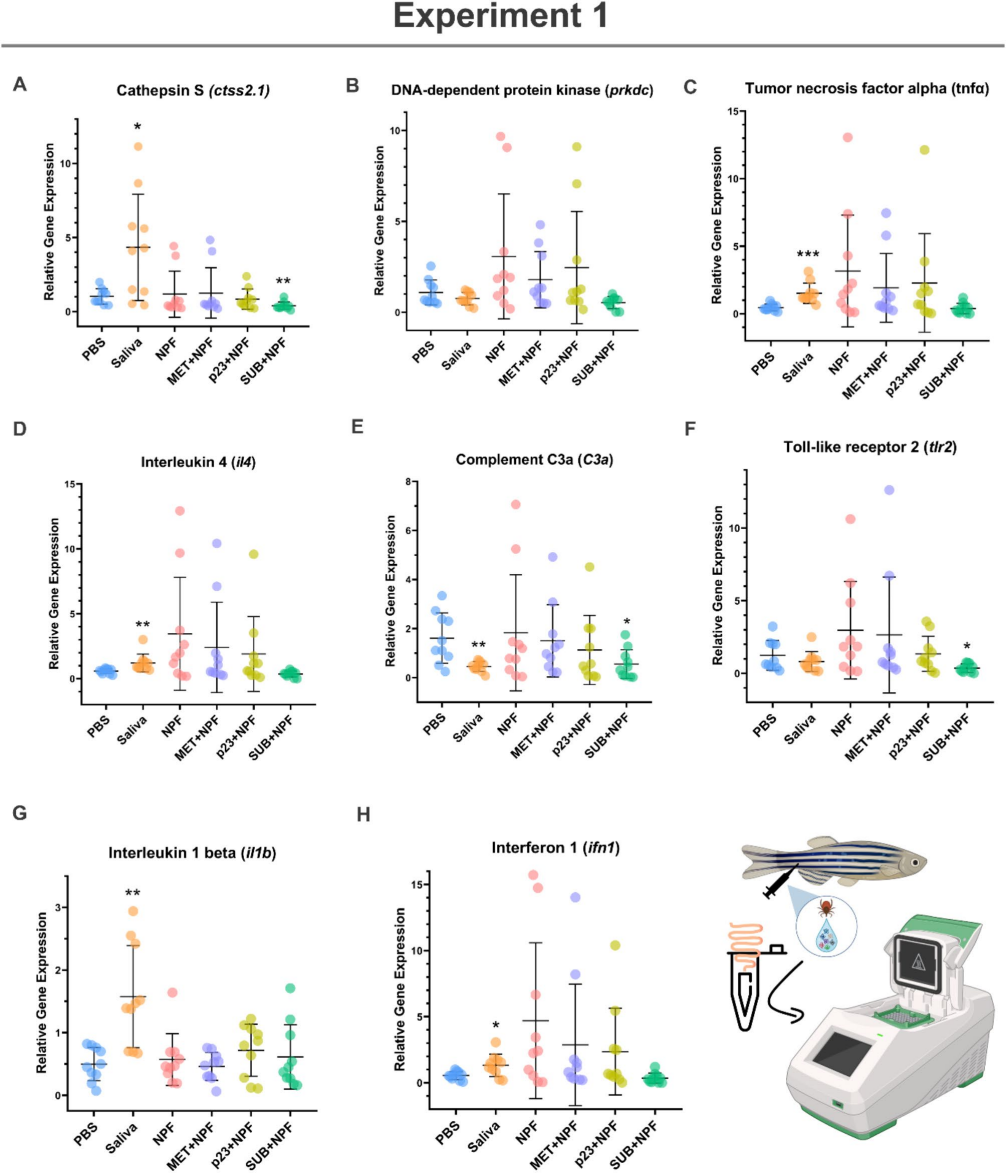
Experiment 1: Expression levels of different zebrafish gut immunity and allergy biomarkers. Expression levels were characterized in zebrafish samples collected from Experiment 1 (Fig. 1, Experimental design to characterize the role of tick salivary proteins in allergic reactions to mammalian meat consumption in the zebrafish model of the α-Gal syndrome). The mRNA levels for (**A**) Cathepsin S, (**B**) DNA-dependent protein kinase, (**C**) Tumor necrosis factor alpha, (**D**) Interleukin 4, (**E**) Complement C3a, (**F**) Toll-like receptor 2, (**G**) Interleukin 1 beta and (**H**) Interferon 1 were analyzed by quantitative reverse transcription polymerase chain reaction (RT-qPCR). Significant differences between groups were analyzed using the Real Statistics extension for Microsoft Excel (v2310) and data was depicted with GraphPad Prism (version 8.0.1) (**p < 0.01).

**Fig. 8:**
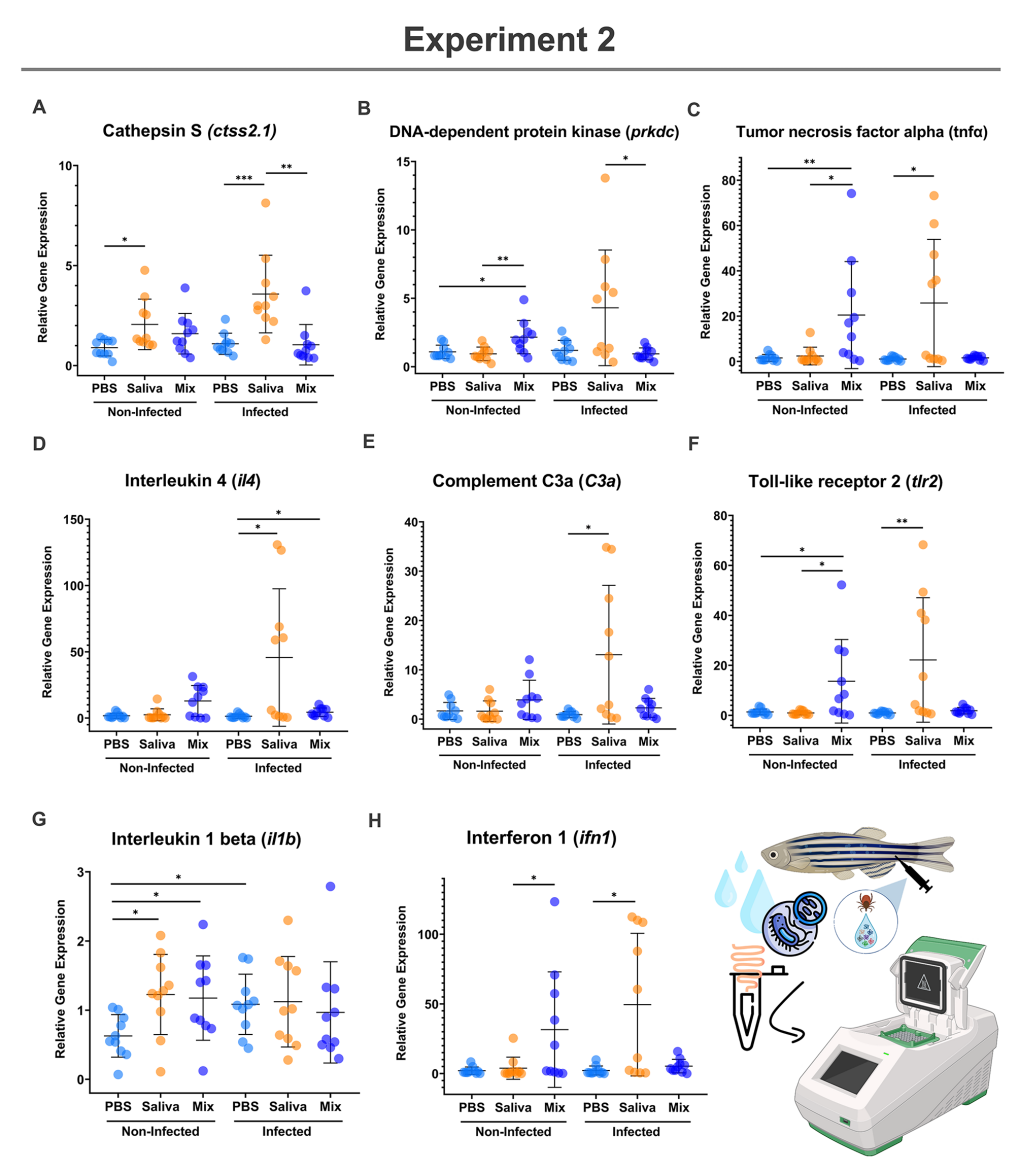
Experiment 2: Expression levels of different zebrafish gut immunity and allergy biomarkers. The mRNA levels were analyzed by quantitative reverse transcription polymerase chain reaction (RT-qPCR). Expression levels were characterized in zebrafish samples collected from Experiment 2 (Fig. 3, Experimental design to evaluate the role of combined treatment with tick salivary proteins and fish tuberculosis). The mRNA levels for (**A**) Cathepsin S, (**B**) DNA-dependent protein kinase, (**C**) Tumor necrosis factor alpha, (**D**) Interleukin 4, (**E**) Complement C3a, (**F**) Toll-like receptor 2, (**G**) Interleukin 1 beta and (**H**) Interferon 1 were analyzed by quantitative reverse transcription polymerase chain reaction (RT-qPCR). Significant differences between groups were analyzed using the Real Statistics extension for Microsoft Excel (v2310) and data was depicted with GraphPad Prism (version 8.0.1) ((* *p* < 0.05, ** *p* < 0.01, *** *p* < 0.001).

The mRNA levels of selected biomarkers associated with immune response and allergic reactions were evaluated in zebrafish gut samples from Experiments 1 and 2 (Figs. 7A and H and 8A and H; Table 1).

**Table 1:** Immune response and allergy biomarkers significant P value results (*p* < 0.05) for each experimental group compared to the PBS control group.

Experiment 1								
Target								
Group	*ctss2.1*	*prkdc*	*tnfα*	*il4*	*C3a*	*tlr2*	*il1b*	*ifn1*
Tick saliva	0.03*	NS	0.0003***	0.001**	0.005**	NS	0.003**	0.01*
NPF	NS	NS	0.07-NS	0.08-NS	NS	NS	NS	NS
MET+NPF	NS	NS	0.05-NS	NS	NS	NS	NS	NS
p23+NPF	NS	NS	NS	NS	NS	NS	NS	NS
SUB+NPF	0.001**	NS	NS	0.07-NS	0.01*	0.02*	NS	NS
Experiment 2								
Target								
Group	*ctss2.1*	*prkdc*	*tnfα*	*il4*	*C3a*	*tlr2*	*il1b*	*ifn1*
Tick saliva	0.02*	NS	NS	NS	NS	NS	0.01*	NS
Mix (MET+p23+NPF)	NS	0.02*	0.009**	NS	NS	0.04*	0.04*	0.08-NS
Saliva+Inf	0.0004***	0.08-NS	0.02*	0.01*	0.03*	0.007**	NS	0.02*
Mix+Inf	NS	NS	NS	0.01*	NS	NS	NS	0.06-NS

*C3a* - complement C3a (NM_131243.1), *ctss2.1 -* cathepsin S (NM_001024409.2), *ifn1* - interferon 1 (NM_207640.2), *il1b* - interleukin 1 beta (NM_212844), *il4* - interleukin 4 (NM_001170740.1), Inf – challenge with *Mycobacterium marinum* (50 ± 8 CFU/mL); NPF – non-protein tick salivary fraction, NS – non significant, MET - metalloprotease (A0A0K8RCY8), Mix - combination of recombinant tick salivary proteins, metalloprotease and allergen-like p23, with saliva non-protein fraction, p23 - allergen-like p23 (A0A0K8RKR7), *prkdc* - DNA-dependent protein kinase (XM_009303401.2), SUB – subolesin (Q4VRW2), *tlr2* - toll-like receptor 2 (NM_212812.1), *tnfα* - tumor necrosis factor alpha (BC165066.1).

In Experiment 1, pro-inflammatory cytokines tumor necrosis factor alpha (*tnfα*) and interleukin 1 beta (*il1b*) were significantly overexpressed in zebrafish injected with tick saliva (*p* < 0.01) when compared to the PBS control group (Fig. 7C and G). Furthermore, *tnfα* also presented an overexpression trend in groups injected with NPF (*p* = 0.07) and recombinant tick protein MET with NPF (*p* = 0.05). Type I interferon response (*ifn1*) was also higher in zebrafish treated with tick saliva (*p* < 0.01, Fig. 7H). Allergic inflammatory biomarkers, cathepsin S (*ctss2.1*) and interleukin 4 (*il4*), exhibited significant higher levels in group inoculated with tick saliva (*p* < 0.05) relative to the PBS-control group (Fig. 7A and D). Both allergic biomarkers (*ctss2.1*, *p* < 0.001 and *il4*, *p* = 0.07) presented lower or similar expression levels, respectively in zebrafish treated with SUB combined with NPF when compared to the PBS-control group (Fig. 7A and D). Lower mRNA levels were also observed in SUB + NPF group for the innate immune inductor, toll-like receptor 2 (*tlr2*, *p* < 0.05, Fig. 7F). Inflammatory mediator, complement C3a (*C3a*), showcases lower values in tick saliva and SUB + NPF groups than in the PBS-control group (*p* < 0.05, Fig. 7E). No significant differences were found between groups (*p* > 0.05) in the genetic marker DNA-dependent protein kinase (*prkdc*) involved in the innate immunity response and pro-inflammatory signaling (Fig. 7B).

In Experiment 2, pro-inflammatory signaling was observed in non-infected zebrafish treated with the combination Mix via significant overexpression of *tnfα*, *il1b*, *tlr2* and *prkdc* (*p* < 0.05; Fig. 8B, C, F and G). The innate immune biomarkers *tnfα*, *ifn1*, *tlr2* and *prkdc* presented higher levels in Mix treated group when compared to non-infected zebrafish treated with tick saliva (*p* < 0.05; Fig. 8B, C, F and H). Treatment with tick saliva in non-infected groups led to the increase of allergic inflammatory mediator *ctss2.1* and pro-inflammatory cytokine *il1b* when compared to PBS control group (*p* < 0.05; Fig. 8A and G).

Zebrafish water-borne challenged with *M. marinum* and treated intramuscularly with tick saliva showed significant higher gene expression of several pro-inflammatory mediators (*tnfα* and *C3a*), allergic biomarkers (*ctss2.1* and *il4*) and innate immune inductors (*tlr2* and *ifn1*) when compared to the infected PBS control group (*p* < 0.05; Fig. 8A, C-F and H). Moreover, expression levels of pro-inflammatory cytokine *il1b*, playing an important role in the control of mycobacterial infection^24^, were significantly hgher in challenged zebrafish treated with PBS only (*p* < 0.05, Fig. 8G). Only cytokine *il4* (type II immune response promoter) was higher in Mix group when compared to PBS control (*p* < 0.05, Fig. 8D). Differences in biomarkers *ctss2.1* and *prkdc* were also observed between tick saliva and Mix groups (*p* < 0.05), with overexpression in zebrafish challenged with *M. marinum* and treated with tick saliva (Fig. 8A and B).

## Discussion

Based on published information, this is the first study that analyzes the effect of tick salivary proteins, MET and p23, in the AGS and tuberculosis zebrafish model to advance in the understanding of associated immunological mechanisms. Our results reveal involvement of tick proteins MET and p23 in zebrafish allergic symptomatology after mammalian meat consumption, driven by pro-inflammatory inductors (*prkdc* and *tlr2*) and cytokines (*tnfα* and *il1b*). The infection with *M. marinum* activated Th1-mediated protective mechanisms reducing Th2 allergic AGS-related symptoms in zebrafish inoculated with tick salivary components and fed on mammalian meat. Furthermore, MET and p23 tick proteins reduced *M. marinum* gut infection levels through protective anti-α-Gal IgM antibodies and lower expression of pro-inflammatory cytokines and mediators (*tnfα*, *il1b*, *tlr2* and *prkdc*).

As previously reported in the zebrafish model of AGS^21,22^, treatment with tick saliva resulted in significant higher incidence of abnormal feeding behavior and/or mortality. The effect of treatment with tick salivary proteins MET and p23 in combination with NPF to mimic saliva on zebrafish hemorrhagic type allergic reactions and mortality was in accordance with saliva protein fractions in which these proteins were identified by mass spectrometry (fractions 3 and 4)^22^. However, after challenge with *M. marinum*, the causative agent of mycobacteriosis in the zebrafish model of tuberculosis^19,20,25,26^, results suggested a protective effect of infection on AGS-related symptoms in response to tick salivary components and mammalian food consumption. These findings are supported by the several protective benefits attenuated mycobacteria have proven to possess in homologous^27^ and heterologous^28^ infections. Nevertheless, although immune modulation caused by inactivated mycobacteria inhalation has proven to ameliorate allergic asthma^29^, these results are the first evidence of benefits related to the attenuation of AGS symptomatology in response to mycobacterial infection. In this case, humoral antibody response and cytokine *il1b* expression in *M. marinum*-challenged zebrafish may be the immune protective modulators. Accordingly, a study revealed that suppression of the allergic Th2-mediated response in latent tuberculosis leads to a shift in Th1 and inhibition of IL-4 production, thus reducing susceptibility to allergic disorders^30^.

Additionally, treatment with tick salivary biomolecules containing MET and p23 in the zebrafish model of AGS reduced *M. marinum* gut infection levels and risk of tuberculosis, presenting lower values of pro-inflammatory cytokines and mediators (*tnfα*, *il1b*, *tlr2* and *prkdc*), combined with previously reported protective titers of anti-α-Gal IgM antibodies^20^. Higher expression of cytokine *il4* triggered by tick proteins and *M. marinum* infection can help to protect against bacterial and parasitic infections^31^, but may also result in a Th2-sweded immune prolife increasing allergic inflammation and the potential development of AGS^10,32,33^.

These results support further studies to fully characterize the protective mechanisms elicited by tick salivary proteins such as MET and p23 against tuberculosis in the zebrafish model with potential applications to control fish mycobacteriosis^34^ and human tuberculosis^35^.

The immune-mediated effects of tick saliva on the zebrafish model have been reported^21,22^, but this study reports activation of several pro-inflammatory mediators (*tnfα* and *il1b*), allergic inflammatory biomarkers (*ctss2.1* and *il4*) and innate immune inductors (*tlr2* and *ifn1*), and inhibition of host alternative complement pathway (*C3a*) crucial for tick feeding^36–38^. Recombinant tick proteins MET and p23 in conjunction with NPF activated the expression of pro-inflammatory inductors (*prkdc* and *tlr2*) and cytokines (*tnfα* and *il1b*) in zebrafish gut. Genetic upregulation of *prkdc* is present in the inflammatory allergic signaling pathways of diseases such as asthma^39^ and the activation of several toll-like receptors contribute to the enhancement of allergic airway inflammation^40^. Moreover, pro-inflammatory cytokines also play a role as enhancers of allergic inflammation^41,42^ and are involved in the AGS pathophysiology^43^.

**Fig. 9:**
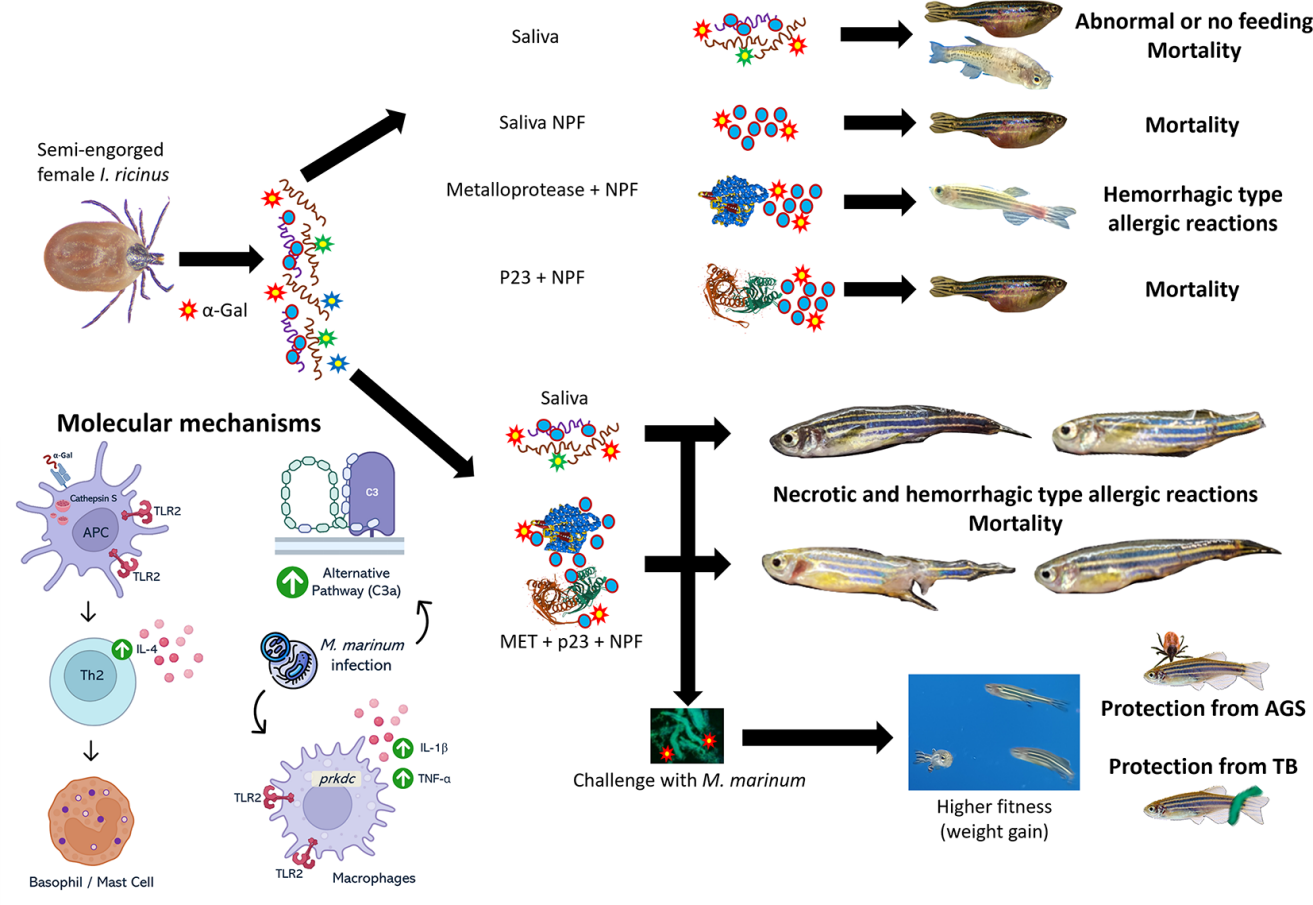
Summary of results. Allergic type reactions to tick salivary compounds and protective mechanisms in response to mycobacteria. Summarized immune effects and molecular mechanismsin zebrafish gut caused by *I. ricinus* tick saliva treatment and water-borne exposure to *M. marinum*. Injection of *I. ricinus* tick saliva containing the glycan α-Gal and proteins like metalloproteases and p23 activates antigen presenting cells (APCs) controlled by the overexpression of cathepsin S (*ctss2.1*) and other immune modulators. APCs charged with toll like receptors 2 (TLR2) activate Th2 responsive cells that produce interleukin 4 (IL-4), which leads to basophil and mast cell recruitment. This type of response is common in allergic inflammation and parasitic infection. On the other hand, bacterial infection by *M. marinum* is responsible for the activation of alternative pathway of complement, measured through the genetic expression of *C3a*. Furthermore, a Th1 driven response is present, marked by TLR2 signaling, activation of pro-inflammatory mediators like DNA-dependent protein kinase (*prkdc*) and cytokines such as interleukin 1 beta (IL-1β) and tumor necrosis factor alpha (TNF-α).

In conclusion, this study reflects potential involvement of tick salivary proteins MET and p23 in response to α-Gal besides the known influence that this glycan has in the AGS pathophysiology^3,44,45^ (Fig. 9). These findings suggest future research to promote the development of diagnostic, therapeutic and preventive measures to lessen the AGS impact on human health. Results suggesting that infection with mycobacteria reduces the allergic susceptibility on the AGS zebrafish model provide insights into the catastrophic selection events and how to further explore these protective mechanisms. On the other hand, the use of tick salivary proteins may also play an important role in reducing *M. marinum* infection levels (Fig. 9). Additionally, deepening the immunological knowledge regarding the AGS and *M. marinum* infection is crucial and lead the identification of novel antigens, potentially developing effective vaccines or personalized treatments.

## Methods

### Ethics statements

As previously reported^22^, experiments were conducted in the zebrafish model of AGS in strict accordance with the recommendations of the European Guide for the Care and Use of Laboratory Animals. Fish were housed and experiments conducted at an experimental facility (IREC, Ciudad Real, Spain) with the approval and supervision of the Ethics Committee on Animal Experimentation of the University of Castilla-La Mancha (PR-2021-09-14) and the Counseling of Agriculture, Environment and Rural Development of Castilla-La Mancha (REGA code ES130340000218). The pathogen-free *I. ricinus* saliva samples were obtained from the Institute of Parasitology, Biology Centre of the Czech Academy of Sciences of the Czech Republic (IPBCAS), in České Budějovice. Semi-engorged female ticks fed for 6–7 days on guinea pigs were treated with pilocarpine hydrochloride (Sigma-Aldrich, St. Louis, USA) and saliva was collected as previously described^21,46^. These animal experiments were performed in accordance with the Animal Protection Law of the Czech Republic No. 246/1992 Sb (ethics approval No. 34/2018). The study is reported in accordance with ARRIVE guidelines (https://arriveguidelines.org).

### Experimental design

Two experiments were conducted in the zebrafish model of AGS^21,22^ and tuberculosis^19,20^. Experiment 1 was designed to characterize the role in AGS of *I. ricinus* tick salivary proteins MET and p23 associated with hemorrhagic type allergic reactions, abnormal behavior pattern and mortality in response to mammalian meat (dog food) consumption^22^ (Fig. 1). Tick intracellular regulatory protein SUB was used as negative control. Tick salivary proteins MET and p23 do not contain α-Gal or its content is unknown, respectively^22^. Accordingly, recombinant proteins (5 µg) were administered in combination with NPF (1 µl) in 10 µl PBS to mimic tick saliva for treatment. Separate treatments with 10 µl of each PBS, tick saliva (1:10) and NPF (1:10) were also included. Experiment 2 was designed to evaluate the role of combined treatment with MET-p23 (2.5 µg of each protein) and NPF (1 µl) in 10 µl PBS (Mix) and dog food consumption on AGS (hemorrhagic type allergic reactions and mortality) and fish tuberculosis (infection by water borne exposure with 50 ± 8 CFU/mL of α-Gal-containing pathogen, *M. marinum*) (Fig. 3). Separate treatments with 10 µl of each PBS and tick saliva (1:10) were also included. In both experiments, saliva from semi-engorged *I. ricinus* female ticks was collected and used for treatment and to prepare the NPF for use alone and in combination with tick recombinant proteins. Wild type adult (6-8-month-old) AB strain zebrafish (20–30 animals per group; 1:1 female to male ratio; 0.33 ± 0.06 g and 0.32 ± 0.08 g for Experiments 1 and 2, respectively) were kept on fish feed during pre-treatment and until Day 2. At Days 0 and 3, zebrafish were intramuscularly injected with each treatment and from Day 2 and until the end of the experiment at Days 8 (Experiment 1) or Days 7 and 25 (Experiment 2) fish were fed with dog food containing mammalian meat. Zebrafish local hemorrhagic type allergic reactions (skin redness), behavior patterns, abnormal or no feeding and accumulated mortality were examined daily throughout the experiment and compared between treatments from Day 1 and until end of the trial using a one-way ANOVA test with post-hoc Tukey Honestly Significant Difference (HSD) test (*p* < 0.05) and the freely available online web calculator Astatsa^47^, as previously reported^21,22^. At the end of the trial, fish were weighted to evaluate weight gain and compared with PBS-treated group by Student’s t-test (*p* < 0.05) using the Real Statistics extension for Microsoft Excel (v2310). Fish euthanasia was performed by prolonged immersion using an overdose of tricaine methane sulfonate (MS222, 200–300 mg/l)^22,48^. Afterwards, serum was collected individually to determine IgM antibody titers against tick salivary glands protein extract, α-Gal and inoculated tick proteins MET and p23. Moreover, IgM levels against SUB for Experiment 1 and *Mycobacterium bovis* P22, an immunopurified subcomplex protein from bovine tuberculin purified protein derivative [bPPD], for Experiment 2 were also measured. Tissue samples from intestine, an organ involved in innate and adaptive fish immunity, were collected and used for analysis of expression of selected immune response gene markers by quantitative reverse transcription polymerase chain reaction (RT-qPCR). In Experiment 2, *Mycobacterium marinum* DNA levels were analyzed by quantitative polymerase chain reaction (qPCR) using zebrafish intestine samples.

### *Ixodes ricinus* tick saliva and non-protein fraction (NPF)

Semi-engorged female ticks fed for 6–7 days on guinea pigs were treated with pilocarpine hydrochloride (Sigma-Aldrich, St. Louis, USA) and saliva was collected as previously described^21,46^. Saliva was then transported and stored at -80 ºC until used. Tick saliva (9 µl) was diluted 1:20 in PBS and 180 µl were ultrafiltrated through Amicon 3 kDa Ultra-0.5 Centrifugal Filter Devices (Merck & Co., Kenilworth, USA) in a centrifuge for 30 min at 14,000 x g. Of them, 120 µl passed through Amicon membrane and were considered the NPF. The α-Gal content in NPF previously determined by ELISA was 0.04 ng/µg proteins compared to 1.1 in saliva protein fraction^22^. The α-Gal modifications may be present in peptides with less than 3 kDa or in glycolipids^49^.

### Production of Recombinant *I. ricinus* tick proteins

Metalloprotease (MET, A0A0K8RB81_IXORI) was synthesized as recombinant protein (Genscript Corporation, Piscataway, NJ, USA). Allergen-like p23 (p23, A0A0K8RKR7), was amplified from a synthetic gene optimized for codon usage in *Escherichia coli* (Genscript Corporation, Piscataway, NJ, USA). Both this protein and subolesin (SUB, Q4VRW2) were produced in *Escherichia coli*. The amplified DNA fragment from p23 protein was cloned into the expression vector pET101 using the Champion pET101 Directional TOPO Expression kit (Carlsbad, CA, USA) and both proteins were expressed in *E. coli* strain BL21, as previously described^50^. Recombinant proteins were fused to Histidine tags for purification by affinity to Ni in the presence of 7 M urea lysis buffer^50,51^. The purified antigens were refolded by dialysis against 1,000 volumes of PBS, pH 7.4 (137 mM NaCl, 2.7 mM KCl, 10 mM Na2HPO4, 1.8 mM KH2PO4) for 12 h at 4 °C.

### Zebrafish

As in previous studies^21,22^, wild type adult (6-8-month-old) AB male and female zebrafish were provided by Dr. Juan Galcerán Sáez from the Instituto de Neurociencias (IN-CSIC-UMH, Sant Joan d’Alacant, Alicante, Spain) and certified by Biosait Europe S.L. (Barcelona, Spain; https://biosait.com) as free of major fish pathogens. Zebrafish were maintained in a flow-through water system at 27 ºC with a light/dark cycle of 14 h/10 h and fed twice daily at 9:30 am and 1:30 pm with dry fish feed (Premium food tropical fish, DAPC, Valladolid, Spain; 50–70 µg/fish). On Day 2 and until the end of the experiment at Day 8, fish were fed with dog food (Classic red, ACANA, Champion Petfoods LP, Edmonton, Canada; 150–200 µg/fish). Fish feed did not contain any meat or byproducts and was primarily composed of cereals and fish byproducts. Dog food consisted of 16% raw beef and 15% lamb meal. Detailed composition of both fish feed and dog food are provided in previous zebrafish studies^21,22^.

### Challenge with *Mycobacterium marinum* in experiment 2

At day 7 of Experiment 2, zebrafish (*n* = 20/group) were challenged with *M. marinum* (50 ± 8 CFU/mL) via water-borne exposure by immersion for 30 min in 500 mL of water^19,25^. From the intestinal samples obtained at Day 25 (*n* = 15–17/group), total DNA was extracted using the AllPrep DNA/RNA/Protein Kit (Qiagen, Hilden, Germany) and following manufacturer´s instructions. Both concentration (ng/µL) and purity of DNA samples were assessed by quantification of the nucleic acids at an optical density of 260 nm (OD260) and the ratio of absorbance at 260/280 nm, using a Nanodrop One spectrophotometer (Thermo Scientific, Waltham, USA). At the end, concentrations were standardized at 50 ng/µL and all samples were stored at − 80 °C. Amplification of *M. marinum* 16 S ribosomal RNA (*16 S rRNA*) to determine infection levels was realized using the CFX96 real-time PCR detection system (Bio-Rad, Hercules, USA) and the quantification was performed by utilizing SYBR green chemistry (Power SYBR Green, Applied Biosystems, Waltham, USA), following manufacturer’s recommendations. The specific PCR primers (10 µM working solution) used for *16 S rRNA* gene fragment (GenBank accession no. AF456240.1) were forward (16 S-F) 5´-ACT GAG ATA CGG CCC AGA CT-3´, and reverse (16 S-R) 5´- TCA CGA ACA ACG CGA CAA AC -3´ (Sigma-Aldrich). Quantitative PCR (qPCR) conditions comprised an initial denaturation step at 95 ◦C for 1 min, amplification by 40 cycles of 95 ◦C for 20 s and 58 ◦C for 1 min, followed by a dissociation curve analysis. Each qPCR reaction had 2 technical replicates/sample (75 ng/well) and 2 negative controls. Expression of *M. marinum* was normalized with the Delta-Delta Ct method (2^−ΔΔCt^), employing as housekeeping gene glyceraldehyde 3-phosphate dehydrogenase (*gapdh*; GenBank accession no. NM_001115114.1; Supplementary Table 1). Finally, to assess statistical significance, the non-parametric Kruskal Wallis test with the subsequent pairwise Mann–Whitney post-hoc test was performed using the Real Statistics extension for Microsoft Excel (v2310) and graphically represent with GraphPad Prism (version 8.0.1).

### Characterization of IgM antibody titers in zebrafish

Indirect in-house ELISAs were conducted in Experiment 1 and 2 to measure IgM antibody titers against tick salivary gland protein extract, α-Gal (#NGP0203; Dextra, Shinfield, UK), p23 (A0A0K8RKR7) and MET (A0A0K8RCY8). Furthermore, anti-SUB and anti-*M. bovis* P22 protein complex IgM antibody titers were measured exclusively for Experiment 1 and 2, respectively. Tick salivary gland protein extracts were obtained from salivary glands of eight female *I. ricinus* ticks-pool fed for 5 days^22^. A BCA protein assay (Bio-Rad, Hercules, USA) was used for total and target recombinant tick protein quantification.

Detailed ELISA protocols can be found in previous zebrafish studies^21,22,25^. Briefly, plates were coated with 50 ng/well of tick salivary gland proteins or 100 ng/well of target protein (p23 or MET) diluted in carbonate-bicarbonate buffer (Sigma-Aldrich, St. Louis, USA) and incubated overnight at 4 °C with gentle shaking. After 3 washes with 200 µl/well PBS with 0.05% Tween 20 (PBST) (Sigma-Aldrich), coated plates were blocked with 100 µl/well of 5% skim milk (Condalab, Madrid, Spain) in PBS (blocking solution) for 1 h at room temperature (RT). Then, 100 µl/well of zebrafish serum samples were diluted in blocking solution (1:100, v/v) and incubated for 1.5 h at 37 °C. Plates were washed 3 times with PBST and 100 µl/well of rabbit anti-zebrafish IgM antibodies diluted in blocking solution (1:1,000, v/v) was added and incubated for 1 h at RT. After 3 more PBST plate washes, 100 µl/well of goat anti-rabbit IgG-peroxidase conjugate (Sigma-Aldrich) diluted in blocking solution (1:3,000, v/v) was added and incubated for 1 h at RT. Final plate washes were performed and incubation with 100 µl/well of 3,3´,5 × 50-tetramethylbenzidine (TMB) One Solution (Promega, Madison, USA) was done for 15–20 min at RT in the dark. Reactions were stopped with 50 µl/well of 2 N H_2_SO_4_ and the optical density (OD) measured at 450 nm in a spectrophotometer (Multiskan, Thermo Fisher Scientific). Antibody levels (OD_450_) were used for analysis after background with blank subtraction (coated wells incubated with PBS). Results were analyzed with the one-way ANOVA test and subsequent Tukey’s HSD post hoc test using the Real Statistics extension for Microsoft Excel (v2310) and graphically represent with GraphPad Prism (version 8.0.1).

### Expression of selected immune response and allergy biomarkers by RT-qPCR

Zebrafish intestinal tissue samples collected during fish necropsy were used to extract mRNA. Firstly, up to 30 mg of tissue/sample was disrupted with a scalpel and homogenized with a tissue grinder until lysates were completely clear. Total mRNA was isolated from 120 samples (10 samples/group from Experiment 1 and 2) using the AllPrep DNA/RNA/Protein Kit (Qiagen, Hilden, Germany) and following manufacturer’s instructions. Both concentration (ng/µL) and purity of mRNA samples were assessed using a Nanodrop One spectrophotometer (Thermo Scientific, Waltham, USA). At the end, concentrations were standardized at 50 ng/µL and all samples were stored at − 80 °C. The mRNA levels of selected immune response and allergy biomarkers^19–21,39,52–54^ were analyzed by quantitative reverse transcription polymerase chain reaction (RT-qPCR) employing the CFX96 real-time PCR detection system (Bio-Rad, Hercules, USA) and gene-specific primers (Supplementary Table 1). Selected zebrafish gene biomarkers included cathepsin S (*ctss2.1*; NM_001024409.2), DNA-dependent protein kinase (*prkdc*; XM_009303401.2), tumor necrosis factor alpha (*tnfα*; BC165066.1), interleukin 4 (*il4*; NM_001170740.1), complement C3a (*C3a*; NM_131243.1), toll-like receptor 2 (*tlr2*; NM_212812.1), interleukin 1 beta (*il1b*; NM_212844) and interferon 1 (*ifn1*; NM_207640.2). RT-qPCR conditions applied included a reverse transcription reaction at 50 ◦C for 10 min, followed by an initial denaturation step at 95 ◦C for 1 min, amplification by 40 cycles of 95 ◦C for 20 s and different annealing temperatures (Supplementary Table 1) for 1 min, followed by a dissociation curve analysis. For a total volume of 20 µl, the PCR mixture contained 1.5 µl (75 ng) of sample mRNA, 10 µl of SYBR Green Master Mix (Bio-Rad), 0.25 µl of iScript reverse transcriptase, 1 µl (10 µM) of forward and reverse primers (Sigma-Aldrich) each and 6.25 µl of nuclease-free water. Each PCR reaction had 2 technical replicates/sample and 2 negative controls. The generalized qBase model was employed to calibrate, normalize and obtain the relative expression values of the targeted biomarkers^55,56^, utilizing β-actin 1 (*actb1*; NM_131031.1) and glyceraldehyde 3-phosphate dehydrogenase (*gapdh*; NM_001115114.1) as housekeeping genes. The non-parametric Kruskal Wallis test was employed to assess statistical significance among all groups and the subsequent pairwise Mann–Whitney post-hoc test was performed for statistical comparison of each treatment group with the PBS-control group. These statistical analyses were done using the Real Statistics extension for Microsoft Excel (v2310) and data was depicted with GraphPad Prism (version 8.0.1).

## Electronic supplementary material

Below is the link to the electronic supplementary material.

Supplementary Material 1

## Data Availability

The data reported in this paper are available within this article and the supplementary information.
