# Publisher Correction: Tick salivary proteins metalloprotease and allergen-like p23 are associated with response to glycan α-Gal and mycobacterium infection

**DOI:** 10.1038/s41598-025-02716-2

**Published:** 2025-06-04

**Authors:** Rita Vaz-Rodrigues, Lorena Mazuecos, Marinela Contreras, Almudena González-García, Marta Rafael, Margarita Villar, José de la Fuente

**Affiliations:** 1 Institute for Game and Wildlife Research, SaBio, IREC-CSIC-UCLM-JCCM, Ronda de Toledo 12, 13005 Ciudad Real, Ciudad Real, Spain; 2 Biochemistry Section, Department of Inorganic, Organic Chemistry and Biochemistry, Faculty of Sciences and Chemical Technologies, University of Castilla-La Mancha, Ave. Camilo José Cela 10, Ciudad Real, 13071 Spain; 3 Department of Veterinary Pathobiology, Center for Veterinary Health Sciences, Oklahoma State University, Stillwater, OK 74078 USA; 4 José de la Fuente, SaBioInstituto de Investigación en Recursos Cinegéticos IREC-CSIC-UCLM-JCCM, Ronda de Toledo 12, 13005 Ciudad Real, Ciudad Real, Spain

Correction to: *Scientific Reports* 10.1038/s41598-025-93031-3, published online 14 March 2025

In the original version of the Article, Figure 8 was a duplication of Figure 7.

The original Article has been corrected.

