# Supplementary material for: Tick salivary proteins metalloprotease and allergen-like p23 are associated with response to glycan α-Gal and mycobacterium infection
Source: Sci Rep. 2025 Mar 14;15:8849. doi: 10.1038/s41598-025-93031-3 (PMC11909269; doi:10.1038/s41598-025-93031-3)
Supplement: Supplementary file 1 — Supplementary Material 1 [file 41598_2025_93031_MOESM1_ESM.pdf]

Tick salivary proteins metalloprotease and allergen-like p23 are associated with response to glycan α-Gal

Rita Vaz-Rodrigues[1], Lorena Mazuecos[2], Marinela Contreras[1], Almudena González-García[1], Marta Rafael[1], Margarita Villar[1,2], José de la Fuente[1,3,*]

[1] SaBio. Instituto de Investigación en Recursos Cinegéticos IREC-CSIC-UCLM-JCCM, Ronda de Toledo s/n, 13005 Ciudad Real, Spain.

[2] Biochemistry Section, Department of Inorganic, Organic Chemistry and Biochemistry, Faculty of Sciences and Chemical Technologies, Universidad de Castilla-La Mancha, Ave. Camilo José Cela 10, 13071 Ciudad Real, Spain.

[3] Department of Veterinary Pathobiology, Center for Veterinary Health Sciences, Oklahoma State University, Stillwater, OK 74078, USA.

* **Corresponding author:** José de la Fuente, SaBio. Instituto de Investigación en Recursos Cinegéticos IREC-CSIC-UCLM-JCCM, Ronda de Toledo s/n, 13005 Ciudad Real, Spain. E-mail: jose_delafuente@yahoo.com / josedejesus.fuente@uclm.es

Supplementary Information

Supplementary Table 1. Oligonucleotide primer sequences and annealing temperatures used for analysing mRNA expression levels of different gut zebrafish (*Danio rerio*) immunity and allergy biomarkers by quantitative reverse transcription polymerase chain reaction (RT-qPCR).

**Target** (NCBI accession no.)	**Amplicon (bp)**	**Oligonucleotide primers (5´-3´)** Forward (F) & Reverse (R)	**Annealing temperature**	**Reference**
ctss2.1 NM_001024409.2	115	F: CGGGTTTATGAGTGACGCCT<br>R: ACGCTGGGATGAACTGTAGC	60 ºC	[1]
prkdc XM_009303401.2	115	F: GCCTGGCTTGTGACGTTGAC<br>R: ACTGCCACTGTGTCTTGGCT	60 ºC	[1]
tnfα BC165066.1	170	F: GCTTATGAGCCATGCAGTGA<br>R: TGCCCAGTCTGTCTCCTTCT	57 ºC	[2]
il4 NM_001170740.1	190	F: GTGAATGGGATCCTGAATGG<br>R: TTCCAGTCCCGGTATATGCT	55 ºC	[2]
c3a NM_131243.1	264	F: ACGCTCTCTGGATTGAAACA<br>R: TGCCTTCTTGCATGGCAATC	56 ºC	[2,3]
tlr2 NM_212812.1	169	F: TGAATGGGTCGAGGAGATTC<br>R: CACAAAGTGCTCCGACAGAA	56 ºC	[2,3]
il1b NM_212844	189	F: GCATGTCCACATATGCGTCG<br>R: GCTGGTCGTATCCGTTTGGA	59 ºC	[2,3]
ifn1 NM_207640.2	258	F: ATGAGAACTCAAATGTGGAC<br>R: TTACACTCGAGGATTGAC	50 ºC	[3]
gapdh NM_001115114.1	259	F: CGTGGTGCCAGTCAGAACAT<br>R: AGTCAGTGGACACAACCTGG	59 ºC	[1,3]
actb1 NM_131031.1	155	F: AAGCTGTGACCCACCTCACG<br>R: GGCTTTGCACATACCGGAGC	62 ºC	[1,4,5]

ctss2.1: cathepsin S, ortholog2, tandem duplicate 1; *prkdc*: protein kinase, DNA-activated, catalytic subunit; *tnfα*: tumor necrosis factor alpha, TNF superfamily, member 2; *il4*: interleukin 4; *c3a*: complement C3a, tandem duplicate 2; *tlr2*: toll-like receptor 2; *il1b*: interleukin 1, beta; *ifn1*: interferon phi 1; *gapdh*: glyceraldehyde-3-phosphate dehydrogenase; *actb1*: beta actin 1.

References

1. Vaz-Rodrigues R, Mazuecos L, Villar M, Contreras M, Artigas-Jerónimo S, González-García A, et al. Multi-omics analysis of zebrafish response to tick saliva reveals biological processes associated with alpha-Gal syndrome. Biomedicine & Pharmacotherapy. 2023;168:115829.

2. Pacheco I, Contreras M, Villar M, Risalde MA, Alberdi P, Cabezas-Cruz A, et al. Vaccination with Alpha-Gal Protects Against Mycobacterial Infection in the Zebrafish Model of Tuberculosis. Vaccines (Basel). 2020;8:195.

3. Contreras M, Pacheco I, Alberdi P, Díaz-Sánchez S, Artigas-Jerónimo S, Mateos-Hernández L, et al. Allergic Reactions and Immunity in Response to Tick Salivary Biogenic Substances and Red Meat Consumption in the Zebrafish Model. Front Cell Infect Microbiol. 2020;10:78.

4. Thiagarajan SK, Mok SY, Ogawa S, Parhar IS, Tang PY. Receptor-Mediated AKT/PI3K Signalling and Behavioural Alterations in Zebrafish Larvae Reveal Association between Schizophrenia and Opioid Use Disorder. Int J Mol Sci. 2022;23:4715.

5. Foo YY, Motakis E, Tiang Z, Shen S, Lai JKH, Chan WX, et al. Effects of extended pharmacological disruption of zebrafish embryonic heart biomechanical environment on cardiac function, morphology, and gene expression. Dev Dyn. 2021;250:1759–77.
